# Subwavelength plasmonic nanoantenna as a Plasmonic Induced Polarization Rotator (PI-PR)

**DOI:** 10.1038/s41598-020-59621-z

**Published:** 2020-02-18

**Authors:** Qaisar Hayat, Junping Geng, Xianling Liang, Ronghong Jin, Khizar Hayat, Chong He

**Affiliations:** 1 Department of Electronics Engineering, Shanghai Jiao Tong University, Shanghai, P.R. China; 2 Department of Physics, Abdul Wali Khan University Mardan, Mardan, Pakistan

**Keywords:** Electrical and electronic engineering, Nanophotonics and plasmonics, Nanophotonics and plasmonics, Photonic devices

## Abstract

This article reports the finding of the plasmonic induced polarization rotation and propagation rotation when the plane EM wave radiates the adjacent active coated nano particle and large dielectric sphere at resonant frequency. The results investigate that the incident electromagnetic planewave excites the TM_21_ mode in the large size dielectric sphere at first, which affects the TM_11_ mode field from the coated nano particle. Consequently, when the combined active coated nano particle and large dielectric sphere are in resonant, the main E–field polarization direction becomes parallel to the propagation direction of the original planewave and main beam of the pattern becomes omnidirectional i.e. both rotates by 90°. Furthermore, the polarization and propagation rotation angle varies with different size of the dielectric sphere. Likewise, the structure of dielectric sphere clamped by two active nanoparticles is also showing plasmonic induced polarization and propagation rotation along with TM_11_ mode from each coated nano particle (CNP) having 180° phase difference. In addition to this, the induced polarization rotation was also verified by the Electric Hertzian Dipole (EHD). The integration of this simpler geometry with other optical devices has possible applications in polarization manipulation, nano-sensors and detectors on nanoscale.

## Introduction

Surface plasmon resonance has attracted more attentions in previous decade due to its wide range of applications in terahertz frequency regime like sensors, amplifiers, information technology, biomedicine, imaging and spectroscopy^1–7^. But the inherently polarization sensitivity problem in optical applications offered more importance to the manipulation of polarization^8^. In addition to this, the importance of manipulating polarization devices is increasing to fulfill the requirments of higher bit rates for succeeding generation in communication systems at optical regime^6,9,10^.

Various techniques have been proposed for realizing the rotation in polarization, such as the use of electro-optical or thermal strain effects. Similarly, some polarization rotators have been reported like, asymmetrical cross-section in Si nanowires^9,11^, using anisotropic or chiral devices to realize circular polarizers and sensors^12^. The problem facing by these polarization rotators is their definite thickness restrictions and relatively bulky configurations. Incorporating these polarization controllers in an ultra-thin device will be of great concern to associate linear and circular polarization with nano-photonic devices and nano sensors^13^.

Fortunately, the interaction of light with surface plasmon in optical regime offers a capable opportunity to manipulate polarization of light in nanoscale. Furthermore, the recent development showed better controlibility over polarization with a vibrant progresses in currently accessible technology according to the essential thickness and the operating bandwidth e.g. in case of plasmonic nanoantennas using crossed resonators^14–16^, gratings from spiral grooves for optical chirality^17^, gammadion-shaped and L-shaped metalic film arrays^18,19^, the chiral assemblies^20,21^, 3D metamaterials^22^ and achievement of 60° rotation of optical light polarization in optical switching^23^.

In this letter, we are proposing a subwavelength size plasmonic nanoantenna capable of manipulating polarization as well as propagation of optical light by 90°. The proposed plasmonic nanoantenna, based on spherical active coated nano particle (CNP), operates at 600 THz resonance frequency. The plasmonic induced polarization rotation in spherical active CNP model is observable in case when this model is placed in the vicinity of a size optimized resonating dielectric sphere. Consequently, the proposed model i.e. the combination of spherical active CNP attached to the resonating dielectric is termed as plasmonic induced polarization rotator (PI-PR) model.

## Results

### Polarization rotation

**Figure 1:**
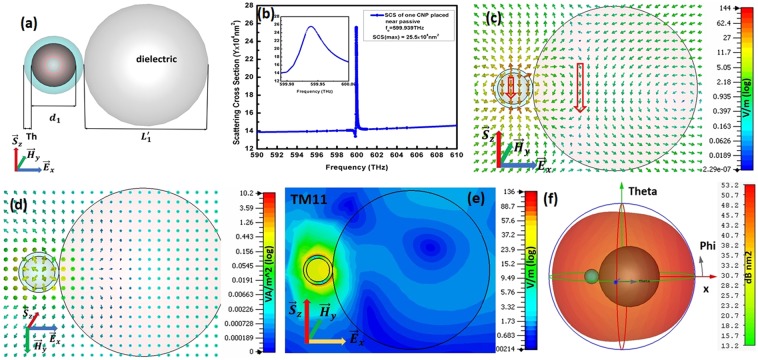
Shows (**a**) basic model of single CNP PI-PR model comprise of spherical active CNP attached to dielectric resonator, (**b**) scattering cross section for single CNP PI-PR model, (**c**) electric field pattern at resonance state, (**d**) power flow, (**e**) TM_11_ mode and (**f**) far field pattern at resonance state.

The basic resonating model of spherical active Coated Nano Particle (CNP) comprise of silver in shell and the silica doped with Er^3+^ fills the core material. A single CNP attached to a dielectric SiO2 constructs the single CNP Plasmonic Induced Polarization Rotator (PI-PR). Figure 1(a) depicts the basic model of the single CNP PI-PR model, where the parameters set ${d}_{1}=46\,nm$ and $Th=6\,nm$ constructs the CNP particle while ${L}_{1}^{{\prime} }=282\,{\rm{nm}}$ is diameter of dielectric attached to CNP. Both the CNP and dielectric resonator have individually matching resonating frequencies i.e. near ${f}_{{\rm{o}}}=600\,{\rm{THz}}$. The resonating frequency for single CNP PI-PR model is ${f}_{{\rm{o}}}=599.939\,{\rm{THz}}$ observable from scattering cross section peak shown in Fig. 1 (b). The xz-cut plane view in Fig. 1 (c) shows the ${\overrightarrow{E}}_{x}$ polarized planewave excites the single CNP PI-PR model and reports $144\,V/m$ electric field. It is recognizable that the rotating electric field in dielectric is rotating the polarization of CNP from ${\overrightarrow{E}}_{x}$ to ${\overrightarrow{E}}_{z}$ direction i.e. in the planewave propagation direction (incident) at resonance frequency ${f}_{o}=599.939\,THz$ and reports TM_11_ mode for CNP attached to dielectric shown in Fig. 1(e). The power flow in Fig. 1 (d) is pointing out from center and flows along x-axis away from the center as represented by the arrows. The far-field pattern shown in Fig. 1(f) also verifies this deviation of planewave propagation from z-axis to x-axis.

### Polarization in individual dielectric and CNP

**Figure 2:**
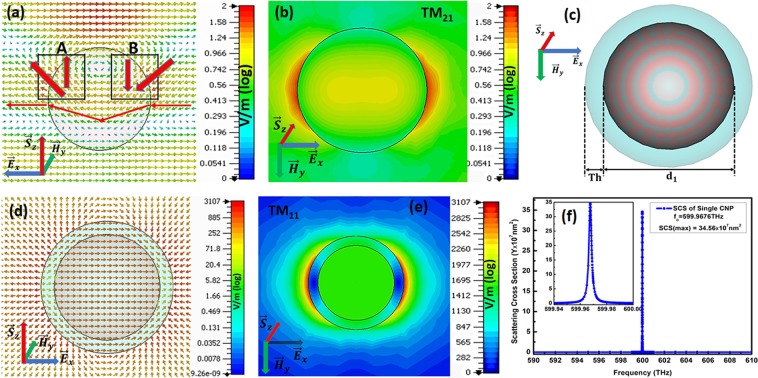
Represent (**a**) electric field pattern for dielectric resonator, (**b**) TM_21_ mode for dielectric resonator, (**c**) basic model of spherical active CNP, (**d**) electric field pattern for single CNP, (**e**) TM_11_ mode for single CNP and scattering cross section for single CNP at resonant frequency respectively.

The electric field pattern of dielectric and spherical active CNP were separately inspected for the enlightenment of polarization rotation. The xz cut plane of dielectric shown in Fig. 2(a) represents the rotation in electric field of ${\overrightarrow{E}}_{x}$ polarized planewave propagating in z direction. When the ${\overrightarrow{E}}_{x}$ polarized planewave propagating in z direction in vacuum refracts into higher refractive index dielectric surface, it bends towards normal and consequently, its electric field also adopts a vertical component. These vertical and horizontal components of electric field are further supported by the electric field of resonant dielectric. Hence enhanced electric field starts circulating inside dielectric due to both horizontal and vertical components. For the optimized size at anticipated frequency (${f}_{0}=600\,THz$), a circular electric field produced inside dielectric is clearly shown by the arrows between A and B in Fig. 2(a). The direction of this circular electric field on left side (near A) of dielectric is parallel to the planewave direction while on right side (near B) it is antiparallel to planewave direction. If a CNP is attached on left or right side of this resonating dielectric, then excitation electric field will already be along z-axis for these CNPs i.e. along positive z-axis on left side while along negative z-axis on right side of dielectric resonator. Similarly, the magnitude of electric field for dielectric is 2 V/m, reported in Fig. 2(a), which is almost double to the incident planewave magnitude. Consequently, this circular radiated electric field will excite the CNPs along z-axis i.e. perpendicular to the original planewave electric field. In addition to this, the yx-cut plane view in Fig. 2(b) reports TM_21_ mode for the dielectric resonator at resonance state. The coated nano-particle model depicted in Fig. 2(c), where silver fills the shell of thickness $Th$ while active silica occupies its core with diameter $\,{d}_{1}$, and hence, it constructs an active spherical coated nano-particle (CNP). Here, the original values are set as $Th=6\,nm$ and ${d}_{1}=48\,nm$. These optimized values of shell thickness and core diameter are the theoretically findings explained in reference^24^. This single spherical active CNP resonates at frequency ${f}_{o}=599.9676\,THz$ as graphed by SCS peak in Fig. 2(f), whereas, xy cut plane view in Fig. 2(e) reports TM_11_ mode for CNP at the mentioned resonating frequency. The electric field arrows in (d) shows the polarization of single CNP along x-axis i.e. in the incident planewave electric field direction.

### Principle for polarization rotation

As discussed in Fig. 2(a) that electric field of planewave adopts two components, i.e. vertical and horizontal components, when refracts from the dielectric surface. The magnitude of these components enhances when dielectric resonates at certain frequency and a circular electric field near center of dielectric is observable. The vertical components on left-hand and right-hand sides of dielectric shown by point A and B in Fig. 2(a) are scattered along z-axis. When the CNP is attached on its either side of dielectric near region A and B this scattered electric field excites CNP along z-axis as observable in Fig. 1(a). The only condition here is that both the resonating dielectric and CNP antenna must have matching resonance frequencies. Here we are in position to propose that for each optimized shell thickness and core diameter (optimized CNP), there exists a size-optimized dielectric resonator whose combine model can constructs a PI-PR at the required optical frequency regime.

**Figure 3:**
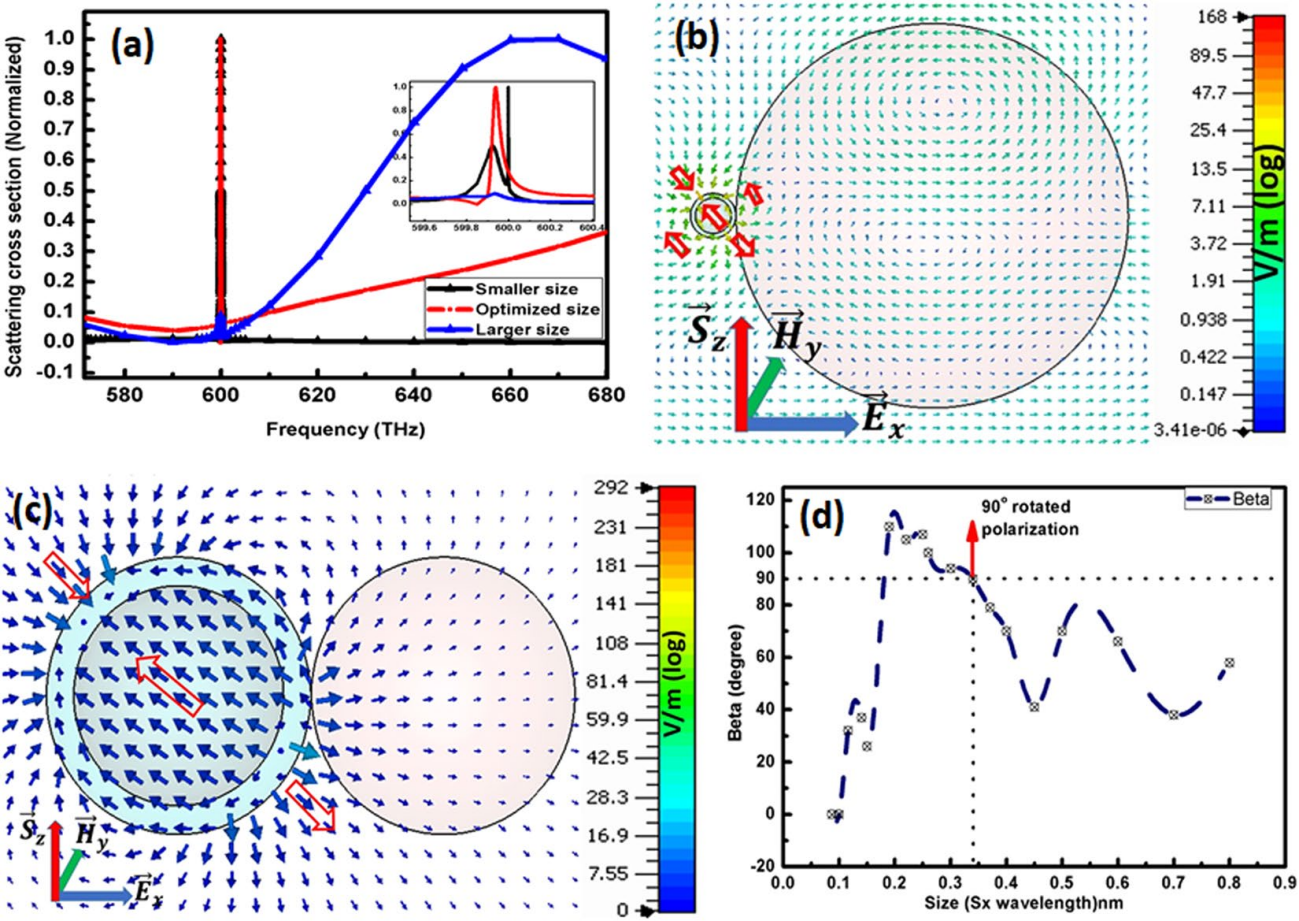
(**a**) Represents normalized scattering cross section of (blue line) higher size of dielectric attached to spherical CNP, (Red line) for optimized size of dielectric and (black line) smaller size of dielectric attached to spherical CNP. Whereas, (**b**) shows electric field for higher dielectric and (**c**) shows electric field for smaller dielectric. Figure 3 (**d**) are the beta vs size relation for single CNP PI-PR model.

The selected size of dielectric i.e. ${L{\prime} }_{1}=282\,nm$ in Fig. 1(a) is resonant near anticipated frequency i.e. ${f}_{o}=600\,THz$. The variation in size of dielectric shifts its resonant frequency i.e. for larger size its resonance state shifts towards higher frequency and vice versa. Blue line in Fig. 3(a) gives an evidence that resonant peak for larger size of dielectric approaches ${f}=660\,THz$ along with small scattering peak for gain medium at ${f}=600\,THz$. Likewise, black line elucidates the lower frequency shift $f=599.89\,THz$ due to smaller size of dielectric i.e. ${L{\prime} }_{1}=58\,nm$ whereas the gain medium peak remains at ${f}=599.938\,THz$. In addition to this, models in Fig. 3(b,c) for these peaks, still reports a feeble rotation of polarization along propagation of planewave in spherical CNPs.

Proceeding Fig. 3(d) portraits the relation of variant sizes vs the angles of the directions difference between the maximum electric field direction of the single CNP case (preceded in Fig. 2(d)) and that of one CNP with dielectric sphere. The resultant radiated electric field is the vector sum of electric fields due to CNP scattering (${\overrightarrow{E}}_{CNP}$), dielectric resonator scattering (${\overrightarrow{E}}_{d}$) and incident planewave (${\overrightarrow{E}}_{in}$). Where ${\overrightarrow{E}}_{d}$ is the effective electric field that can rotate the total scattered electric field. It is perceptible when the dielectric sphere size increases from the optimized size i.e. 0.34λ then there is always rotation of electric field between $0^\circ$ and $90^\circ$ because the resonance of dielectric is reducing and consequently ${\overrightarrow{E}}_{d}$ is reducing. Whereas, for size between 0.18λ to 0.34λ near to resonant size of dielectric, the rotation angle of net electric field is $90^\circ$ ~ $120^\circ$ because size of dielectric resonator is nearby to resonating size, hence ${\overrightarrow{E}}_{d}$ becomes dominant over ${\overrightarrow{E}}_{CNP}$. Moreover, for smaller sizes the dominancy of ${\overrightarrow{E}}_{d}\,$reduces over ${\overrightarrow{E}}_{CNP}$ hence, rotation angle also reduces gradually to $\,0^\circ$. In addition to this, the increase in size from the optimized size of dielectric resonator the scattered radiation starts aligning itself parallel to incident planewave.

### Polarization rotation in two CNPs PI-PR model

**Figure 4:**
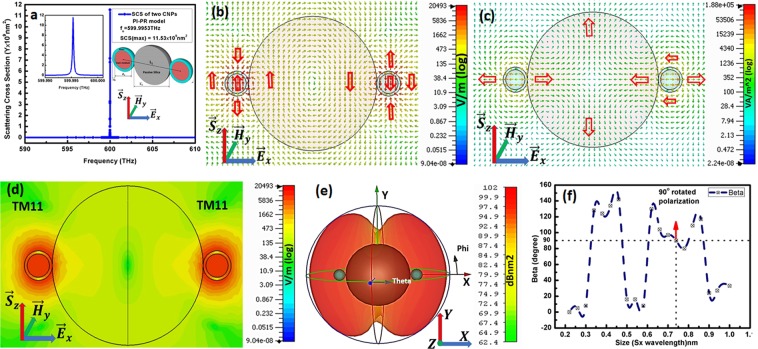
Reports (**a**) two CNPs PI-PR model and SCS at resonance frequency, (**b**) electric field pattern, (**c**) power flow for two CNPs, (**d**) TM_11_ modes (**e**) far field pattern for two CNPs PI-PR model and (**f**) beta vs variant size of dielectrics relation respectively.

We also observe the polarization rotation in case of two CNPs attached to a single dielectric on opposite sides as labeled in Fig. 4(a). The set of parameters of CNPs, for simplified model of the two CNPs PI-PR model, are the same as mentioned for single CNP PI-PR model. The dielectric SiO_2_ of diameter ${L{\prime} }_{1}=313\,{\rm{nm}}$ supplants the empty space between the CNPs for keeping ${L}_{1}=0.742\,{{\rm{\lambda }}}_{0}$ spacing between centers of CNPs.

It is noticeable from electric field distribution in Fig. 4(b) that when a ${\overrightarrow{E}}_{x}$ polarized planewave excites the two CNPs PI-PR model; the polarization rotates to the planewave propagation direction (incident) in both CNPs. Here it is observable that electric field in both CNPs are exactly at $180^\circ$ phase difference with each other. In addition to this, two CNP PI-PR model reports TM_11_ mode shown in (d) for individual CNPs at resonance frequency ${f}_{o}=599.9953\,THz$ represented by SCS peak graphed in Fig. 4(a) where maximum SCS is 11.53 × 109 nm^2^. The power flow shown in Fig. 4(c) is pointing out from center of CNPs and deviates towards x-axis instead of propagating along z-axis as pointed by arrows. The far field pattern in Fig. 4(e) confirms the deviation of energy flow from z-axis to x-axis when the model radiates. The rotation of electric field is elaborated in relation skitched in Fig. 4(f) between variant size of dielectrics and the angles obtained from the directions difference between the maximum electric field direction of the single CNP case (shown in Fig. 2(d)) and that of the left side CNP attached with the dielectric resonator. This deportment is similar to the single CNP PI-PR model as prescribed in Fig. 3(d).

### Excitation of PI-PR by small current

**Figure 5:**
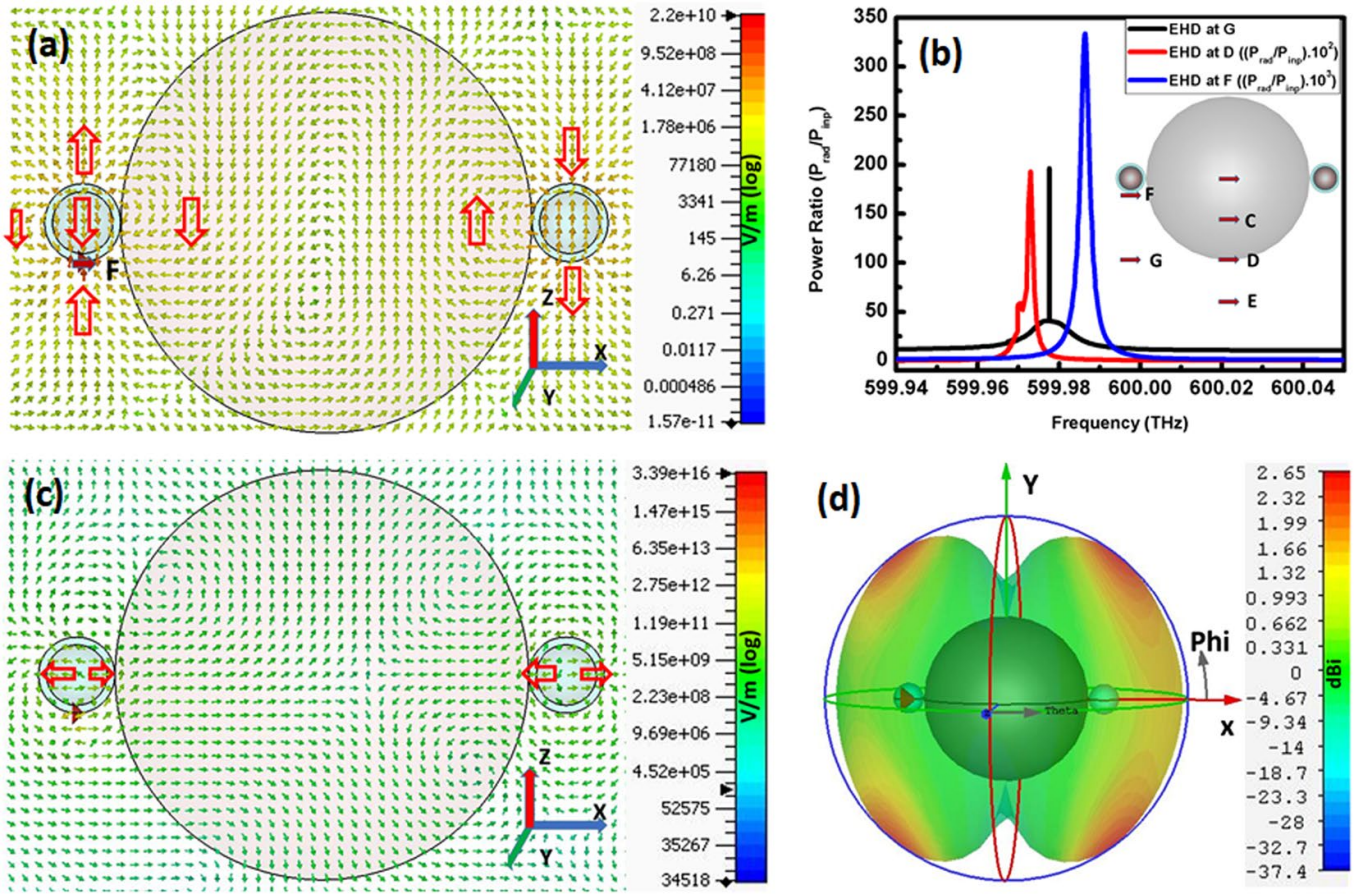
Displays two CNPs PI-PR model excited by EHD where (**a**) displays electric field pattern for EHD at point F while (**b**) displays its power ratio for Points D, F and G. The EHD excitation points C, D, E, F and G are labeled in (**b**). Similarly, (**c**) shows the power flow and (**d**) shows its far field pattern.

Partaking established the polarization rotation in proposed PI-PR models for the planewave excitation, the similar behavior of two CNP PI-PR model is observed under the EHD excitation. Observing the polarization rotation and power ratio, we excite the model by EHD at C, D, E, F and G positions as shown in Fig. 5(b). It is noticeable that whatever the vertical position for EHD excitation is selected the polarization rotates in the direction perpendicular to the external electric field (parallel to propagation direction). Figure 5(b) represents EHD at different possible positions of dielectric/CNP. Results of EHD at center of dielectric are not included here because it has no polarization rotation effect in CNP.

The electric field reported in Fig. 5(a) is $\,2.2\times {10}^{10}\,V/m$ whereas power ratio reported in Fig. 5(b) by blue line for same model has peak value 333450 at ${f}_{o}=599.9864\,THz$. When EHD scans the PI-PR model at surface of CNP along vertical axis (point F in Fig. 5(a)) then it reports maximum power ratio and strong polarization rotation in CNP as compared to other positions. The entire cases for EHD excitation reported in Fig. 5(b) i.e. from C to G, accounts the polarization rotation in CNPs. The power ratio for these aforementioned models at EHD points D, F and G are $PR=193\times {10}^{2},\,334\times {10}^{3}$and $PR=196$ at frequencies ${f}_{o}=599.973\,THz$, $599.9664\,THz$ and $599.9776\,THz$ shown in Fig. 5(b) by red, blue and black curves respectively. In addition to this, the power flow spectrum and far field pattern in Fig. 5(c,d) show the power scattering along x-axis i.e. similar to planewave excitation of two CNPs PI-PR model.

## Discussion

In summary, we present a plasmonic induced polarization rotator that is capable to rotate the polarization and propagation by $\,90^\circ$ at resonate frequency. The plane of polarization rotates perpendicular to the incident electric field and becomes parallel to incident planewave direction. Similarly, the propagation direction deviates by $90^\circ$ and adopts the omnidirection. The rotation of polarization and propagation is due to the inclusion of a spherical dielectric resonator. Both single spherical active coated nano particle attach to a dielectric and a single dielectric sandwiched between two spherical active coated nano particles have the plasmonic polarization rotation for planewave excitation as well as for electric hertzian dipole excitation. Here from electric field pattern, we also investigate that both the spherical active coated nano particle and dielectric needs to be optimized at particular frequency first for a strong plasmonic polarization rotation.

## Methods

### Geometry of antenna model and size effect drude model

This section depicts the design of resonating Active Spherical Coated Nano-Particle (CNP) model, simulated by a computational electromagnetic simulation set of tools utilizing CST Microwave Studio; which solves Maxwell’s equations using frequency domain method. Here we construct two Plasmonic Induced Polarization Rotator (PI-PR) models; a CNP attached with a dielectric (SiO_2_) and a dielectric sandwiched between two CNPs configurations.

### Size dependent drude model

To investigate the optical response of nano-sized metal shell with dielectric core CNP needs to include the size effects in Drude model as the optical response of nano-dimensional materials is tremendously different form their bulk counterpart. The following relation provides the dielectric response of any material in term of Drude Response and interband transition:

1 $${\varepsilon }_{Drude}(R,\omega )=1-\frac{{\omega }_{p}^{2}}{\Gamma (R{)}^{2}+{\omega }^{2}}+j\frac{\Gamma (R{)}^{2}{\omega }_{p}^{2}}{\omega (\Gamma (R{)}^{2}+{\omega }^{2})}$$

Where R, ω_p_ and Γ represents the thickness of the surrounding shell, the plasma frequency and the collision frequency respectively. The values of all Drude parameters and Fermi velocity, used to compute the dielectric dispersion for 6 nm thick silver shell and 24 nm dielectric core are m*/m = 0.96, N = 5.85 × 10^28^ m^−3^, V_F_ = 1.39 × 10^6^ m/sec, ω_p_ = 1.39269 × 10^13^ s^−1^ as given in reference. As the gap of CNPs is enough large to prevent quantum tunneling or charge transfer between CNPs^25,26^ therefore, the size effect in classical Drude model termed here is semi-classical model.

### Lorentz model for gain medium

The CNP comprises of silver shell enclosing gain medium as active core element. This gain medium consists rare earth doped SiO_2_. Here the core material uses the same gain media values for spherical active CNPs. The following relation describes Lorentz Model to incorporate the canonical gain media in CST Microwave Studio:

2 $${\varepsilon }_{r}(\omega )={\varepsilon }_{\infty }+\frac{({\varepsilon }_{s}-{\varepsilon }_{\infty }){\omega }_{0}^{2}}{{\omega }_{o}^{2}+j\omega \Gamma -{\omega }^{2}}$$

The relative epsilon of passive silicon dioxide is set as constant ${\varepsilon }_{r}=2.05$ due to its very low loss characteristics; resulting the refractive index $\,n=\sqrt{{\varepsilon }_{r}}=1.432$.

The optimal value for $\kappa =-\,0.25$ is set for resonance frequency $f={f}_{o}=600\,THz$ (500 nm wavelength) and $\omega ={\omega }_{o}=2\pi {f}_{o}$ to obtain ${\varepsilon }_{\infty }$ and then get ${\varepsilon }_{s}$:

3 $${\varepsilon }_{\infty }=1.9875\,{\rm{and}}\,\frac{({\varepsilon }_{s}-{\varepsilon }_{\infty }){\omega }_{o}^{2}}{\Gamma }=2n\kappa $$

Re-arranging Eq. (6) and replacing ε_∞_ by its value to obtain:

4 $${\varepsilon }_{s}=\frac{2n\kappa \Gamma }{{\omega }_{o}^{2}}+1.9875$$

The collision frequency Γ (damping frequency) is related to the resonance frequency and valued in the range (10^−3^ ~ 10^−1^) ${\omega }_{o}$ to represent low to high loss materials. The spherical active CNP and dielectric SiO_2_ constructs the proposed PI-PR models. The detail study of these material models can be found in^27–31^. The PI-PR models are simulated by a computational electromagnetic simulation set of tools utilizing CST Microwave Studio, which solves Maxwell’s equations using frequency domain method.

### Planewave excitation

The optical deportment of core-shell nano particle are noticeable from scattering cross section (SCS) and absorption cross section (ACS), which can be numerically calculated by Poynting theorem:

5 $${\sigma }_{scat}=\frac{{P}_{scat}}{{I}_{inc}}=\frac{1}{{I}_{inc}}Re\{\frac{1}{2}{\iint }_{s}[{\overrightarrow{E}}_{s}\times {\overrightarrow{H}}_{s}^{\ast }].\hat{n}dS\}\,$$

6 $${\sigma }_{abs}=\frac{{P}_{abs}}{{I}_{inc}}=-\,\frac{1}{{I}_{inc}}Re\{\frac{1}{2}{\iint }_{s}[{\overrightarrow{E}}_{tot}\times {\overrightarrow{H}}_{tot}^{\ast }].\hat{n}dS\,\}$$

where “S” is the surface enclosing the particle and $\hat{n}$ is unit vector, pointing outward the surface. The total SCS and ACS can be defined by ratio of radiated power with incidence irradiance^27,29^. The values of $\,{\sigma }_{scat}$, and ${\sigma }_{abs}$ are radily calculated with the CST post-processing tools.

### Radiated power by small current

The radiated power ratio (PR), i.e., ratio of the total power radiated in the presence of resonating model to its value generated in free space, has been a useful figure of merit for the Electric Hertzian Dipole (EHD) excitations. The total radiated power needs to be considered here to characterize the optical behavior of CNP models excited by EHD. It is related by the Poynting’s vector-based expression:

7 $${P}_{tot}=\mathop{\mathrm{lim}}\limits_{r\to \infty }\frac{1}{2}{\int }_{\theta =0}^{\pi }{\int }_{\varphi =0}^{2\pi }Re\{{\overrightarrow{E}}_{tot}(r,\theta ,\varphi )\times {\overrightarrow{H}}_{tot}^{\ast }(r,\theta ,\varphi )\}\cdot \hat{n}\,dS\,$$

Where the surface S is a sphere enclosing the entire resonating system. For an EHD with a dipole current moment ${p}_{s}={I}_{o}d$ radiating in free space, this yields [21]:

8 $${P}_{i}=\frac{{\eta }_{o}\pi }{3}\,{|\frac{{p}_{s}{k}_{o}}{2\pi }|}^{2}\,$$

The characteristic impedance of free space is ${\eta }_{o}=376.7\,\Omega$. Then the power ratio becomes simply as:

9 $$P{R}_{}=\frac{{P}_{tot}}{{P}_{i}}\,$$
